# Expression of HIF-1α and Markers of Angiogenesis Are Not Significantly Different in Triple Negative Breast Cancer Compared to Other Breast Cancer Molecular Subtypes: Implications for Future Therapy

**DOI:** 10.1371/journal.pone.0129356

**Published:** 2015-06-05

**Authors:** Lamis Yehia, Fouad Boulos, Mark Jabbour, Ziyad Mahfoud, Najla Fakhruddin, Marwan El-Sabban

**Affiliations:** 1 Department of Anatomy, Cell Biology and Physiological Sciences, American University of Beirut, Beirut, Lebanon; 2 Department of Pathology and Laboratory Medicine, American University of Beirut Medical Center, Beirut, Lebanon; 3 Department of Public Health, Weill Cornell Medical College, Doha, Qatar; 4 Department of Pathology, Hammoud Hospital University Medical Center, Sidon, Lebanon; 5 Department of Pathology, Case Western Reserve University School of Medicine, Cleveland, Ohio, 44195, United States of America; University of Dundee, UNITED KINGDOM

## Abstract

**Introduction:**

Triple negative breast cancer lacks estrogen, progesterone and epidermal growth factor receptors rendering it refractory to available targetedtherapies. TNBC is associated with central fibrosis and necrosis, both indicators of tumor hypoxia. Hypoxia inducible factor 1α is up-regulated under hypoxia and its expression is associated with induction of angiogenesis resulting in proliferation, aggressive tumor phenotype and metastasis. In this study we evaluate the potential use of HIF-1α as aTNBC-specific marker.

**Methods:**

62 TNBC, 64 HER2^+^, and 64 hormone-receptors positive breast cancer cases were evaluated for central fibrosis and necrosis, HIF-1α, HIF-1β, VEGFR3, CD31 expression and microvessel density. RNA extraction from paraffin-embedded samples, followed by quantitative real-time polymerase chain reaction (qRT-PCR) evaluation of HIF-1α and VEGF transcripts was performed on 54 cases (18 from each subtype).

**Results:**

HIF-1α protein was expressed in 35.5% TNBC, 45.3% HER2^+^and 25.0% ER^+^/PR^+^ (p = 0.055; χ^2^ test). PCRanalysis of subgroup of breast cancers, 84.2% expressed HIF-1α protein and its transcripts, while only 66.7% expressed VEGF transcripts simultaneously with the HIF-1α protein and its transcripts. Central fibrosis and necrosis was highest in TNBC (p = 0.015; χ^2^ test), while MVD was comparable among all groups (p = 0.928; χ^2^ test). VEGFR3 was highest in TNBC expressing HIF-1α. HIF-1β protein was expressed in 32.0% of HIF-1α(+), and in (44.3%) of HIF-1α(-) breast cancer cases (p = 0.033; χ^2^ test). Moreover, HIF-1α expression in cases with central fibrosis and necrosis was highest in the HER2^+^ followed by the TNBC (p = 0.156; χ^2 ^test).

**Conclusions:**

A proportion of TNBC express HIF-1α but not in a significantly different manner from other breast cancer subtypes. The potential of anti-HIF-1α targeted therapy is therefore not a candidate for exclusive use in TNBC, but should be considered in all breast cancers, especially in the setting of clinically aggressive or refractory disease.

## Introduction

Breast carcinoma exhibits regional hypoxia during its early stages of development. Under hypoxic conditions, the induced “angiogenic switch” causes an elevated expression of Hypoxia inducible factor 1α(HIF-1α) [1–3], followed by “vascular endothelial growth factor (VEGF)-induced angiogenesis”, and consequently tumor vascularization[4], which promotes tumor progression, invasion and eventually metastasis[5–7]. Although high HIF-1α expression was documented in all breast carcinoma subtypes, a stronger correlation was found with non-heritable and heritable *BRCA1* mutation-associated cancers, which in turn are associated with the basal-like molecular subgroup and a triple-negative phenotype[8–10].

Triple negative breast cancer (TNBC), which is defined by the lack of estrogen receptor (ER), progesterone receptor (PR), and human epidermal growth factor receptor 2 (HER2) expression, accounts for 10–17% of all breast carcinomas[11–20]. Though heterogeneous, TNBCs are most commonly high-grade invasive ductal carcinomas that often affect younger patients[11,13,16,19,21], and pursue an aggressive clinical course[16,19,22,23]. TNBC is regrettably excluded from the effective targeted therapy used in luminal and HER2-positive breast carcinomas due to its lack of hormonal and Human Epidermal Growth Factor receptor expression [20,24–28].

The association between HIF-1α and the frequently triple negative familial breast cancer brings forth the possibility of novel targeted therapy for TNBC, namely anti-HIF-1α chemotherapy and related agents. This is especially plausible given the frequent association of TNBC with central necrosis, a surrogate morphologic marker for hypoxia.

In this study, we assessed the expression of HIF-1α and other markers of hypoxia and angiogenesis including VEGF, vascular endothelial growth factor receptor 3 (VEGFR3), and microvessel density (MVD) in TNBC as compared to HER2^+^ and luminal-type breast cancers in order to evaluate the practical potential of using anti-HIF-1α as a therapeutic target for TNBC preferentially to other breast cancer subtypes.

## Materials and Methods

Institutional review board at the American University of Beirut approved the study with waiver of a written patient informed consent.

### Patients and specimens

Pathology reports of patients with breast carcinoma between 2001 and 2011 were accessed from the Pathology Departments at the American University of Beirut Medical Center (AUBMC) and Hammoud Hospital University Medical Center (HHUMC). IRB approval was obtained and no patient consent was required. Patients with no prior chemotherapy, radiotherapy, hormonal therapy, or any form of targeted therapy were selected as follows: all TNBC(64) cases (group 1) were identified and retrieved. They were matched with an equal number of ER^-^/PR^-^/HER 2^+^ (group 2) and ER^+^/PR^+^/HER 2^-^ (group 3) breast carcinomas. Cases were then re-evaluated for histologic subtype, grade, central fibrosis and tumor necrosis, as well as adequacy (two TNBC core biopsies were excluded because of minimal (<200) number of tumor cells). For each of the included one hundred and ninety (190) cases, a representative formalin-fixed paraffin-embedded (FFPE) tissue block was selected for immunohistochemical (IHC) and molecular analyses. Negative controls for both techniques were obtained from reduction mammoplasty tissue. ER, PR, and HER2 expression was assessed using immunohistochemistry according to the ASCO-CAP guidelines 2010 and 2007 respectively[29,30]. In addition, presence of central necrosis and fibrosis was considered when we identified a central scar defined as a central, predominantly acellular area of tumor showing sclerosis, myxoidstroma, fibrosis or necrosis[28].

### Immunohistochemistry

Three sections (3μm thick) per selected paraffin block were prepared and deparaffinized to be stained for HIF-1α(H1a67,Abcam, San Francisco, CA, USA), HIF-1β (ab54786, Abcam, San Francisco, CA, USA), VEGFR3 (KLT9,Novocastra-Newcastle, UK) and CD31(1A10,Novocastra-Newcastle, UK)by immunohistochemistry for all the 190 cases. All antibodies were used at a 1:100 dilution and endogenous peroxidase activity was blocked for 10 minutes in 5% hydrogen peroxide. Antigen retrieval using pepsin was performed for HIF-1α, HIF-1β, and CD31 staining.Biogenics Super Sensitive polymer detection system and DAB (diaminebenzidine) chromogen was used.The slides were then treated in accordance with the manufacturer’s instructions and counter-stained with hematoxylin.

HIF-1α and HIF-1β expression was considered positive when at least 5% of the tumor cells showed nuclear staining[31], and VEGFR3 was considered positive when at least 10% of tumor cells showed cytoplasmic staining [32].

HIF-1α and HIF-1β expression was evaluated by applying the scoring system used by Santos et.al [33]. Each sample was evaluated for intensity of nuclear staining and percentage of positive nuclei. The score for signal intensity is: negative (0), weak (1), moderate (2) and strong (3). The score for percentage of positive nuclei is: (1) when <10% of cells were positive; (2) when 10–50% of cells were positive and (3) when >50% of cells were positive. Then both scores were multiplied, and the HIF1a expression resulting score is designated as negative (<1), weak (1–6) and strong (>6).

### Microvessel density evaluation

Four fields were selected randomly at 20x in the CD31 stained slides, photographed and counted. Microvessels were identified as circumscribed patent lumens surrounded by positively staining endothelial cells. The mean vessel count in all four fields was recorded.

### RNA extraction

#### The RecoverAl

Total Nucleic Acid Isolation Kit (Ambion, Applied Biosystems, California, USA) was used to extract RNA from FFPE breast tissues. 80μm thick ribbons were obtained and RNA extracted according to the manufacturer’s recommendations. RNA was quantified using NanoDrop ND-1000 spectrophotometric system.

### Real-time PCR

54 cases were selected from the 3 groups (18 cases per group) for molecular analysis. The selection aimed to obtain 3 equally distributed categories: ≤40 years, 40 to 60 years, and >60 years of age at diagnosis.

cDNA was synthesized from 1μg of extracted RNA using the RevertAid 1^st^ Strand cDNA synthesis kit (Fermentas). iQ SYBR Green Supermix (Bio-Rad) was used in CFX96 real-time PCR system (Bio-Rad). The cycling conditions included aprecycle for 3 minutes at 95°C, followed by 40 cycles of denaturation (15 seconds at 95°C), annealing (1 minute at the specific primer-optimized annealing temperature), and extension (1 minute at 72°C).Final extension was for 5 minutes at 72°C followed by generating the melting curve from 55°C to 95°C in 0.5°C increments.

The primers used were GAPDH (forward: TGGTGCTCAGTGTAGCCCAG, reverse: GGACCTGACCTGCCGTCTAG) with an annealing temperature of55˚C; HIF-1α (forward: AGCCAGATCTCGGCGAAGT, reverse: CAGAGGCCTTATCAAGATGCG) with an annealing temperature of 58°C; and VEGF (forward: AGGCCCACAGGGATTTTCTT, reverse: ATCAAACCTCACCAAGGCCA) with an annealing temperature of 55°C.

The fluorescence threshold cycle (Ct) value was determined for each gene and normalized with GAPDH. All values were compared and normalized to normal breast tissues.

### Statistical Analyses

Sample characteristics were summarized using means for numeric variables for age and MVD. Frequency distributions were used for the categorical variable VEGFR3. Chi-squared test or Fisher’s exact test (when counts fell below 5) were used to compare VEGFR3 expression and HIF-1α in the three breast carcinoma groups. In addition, Chi-squared test was used to compare HIF-1a with HIF-1β in VEGF positive cases. Pair-wise comparisons were also carried when the differences were significant using the Chi-squared test or Fisher’s exact test. Results of pair-wise comparisons were represented by Roman letters. Groups that were not significantly different were denoted with the same letter. Chi-squared test was used to compare central fibrosis and necrosis. This analysis was also repeated adjusting for age by using multivariate logistic regression.

The PCR data was analyzed by summarizing the fold changes using means and standard deviations along with medians and ranges. Since the distribution of the two variables (HIF-1α and VEGF) was skewed, we compared their median using the Kruskal-Wallis test. The Wilcoxon rank sum test was used to compare the fold change in HIF-1α between those that expressed HIF-1α by immunohistochemistry and those that did not express HIF-1α. Analyses were performed using SPSS software (version 19). A p-value of 0.05 or less was considered statistically significant.

## Results

### Clinical data

Patient age ranged from 26 to 88 years (mean = 52 years, standard deviation = 12.8). Patients younger than 40 years were more prevalent in Groups 1 and 2 (23.3% and 28.1% respectively) (Table 1). More than 80% of TNBC and HER2^+^ groups were grade 3/3 (Nottingham grading system)[34], as compared to 55% in ER^+^/PR^+^ tumors.

**Table 1:** Clinicopathologic variables of TNBC as compared to the HER2+ and ER+/PR+ breast carcinoma.

Clinicopathologic Variables	TNBC (N = 62)	HER2+ (N = 64)	ER+/PR+ (N = 64)
**Age**	52.3	51.7	51.3
**Grade**			
1	3%	3%	6%
2	12%	10%	39%
3	85%	87%	55%
**Central fibrosis and necrosis** *	25.8%^A^	9.4%^B^	10.9%^B^
**Lymph node positive**	45%	53%	44%
**≥4 lymph nodes**	33%	48%	62%
**Microvessel density**	16.5	15.3	14.9

* p = 0.019 indicating a significant difference at the 5% level

A,B; different letters indicate significant differences between groups

### Central fibrosis and necrosis in TNBC

15.3% of all tumors showed central fibrosis and tumor necrosis, which differed significantly among the 3 Groups (p = 0.019) (Table 1). TNBC had the highest values among all groups even after adjusting the results for age (no change in the p-values, results not shown in the table).

### MVD and VEGF expression

MVD showed no significant variation between the 3 groups (Table 1), although the mean MVD count was higher but not significantly so (p = 0.928) in TNBC cases that expressed HIF-1α. In addition, MVD did not correlate with VEGF fold increase significantly (*data not shown*).

HIF-1α nuclear expression by immunohistochemistry was highest in the HER2+ group,howeverthereis no statistical significant difference amongthe three different groups [TNBC (35.5%), HER2^+^(45.3%) and ER^+^/PR^+^(25.0%)] (p = 0.055).

### VEGFR3 expression

VEGFR3 expression differed significantly (p = 0.003) between the three groups with the highest expression in HER2^+^, while the TNBC group had an intermediate value between HER2^+^and ER^+^/PR^+^. Additionally, VEGFR3 expression was higher among patients expressing HIF-1α as compared to those with negative HIF-1α expression, though not in a statistically significant manner.

### Correlation between transcriptional and protein levels of HIF-1α and VEGF

**Fig 1:**
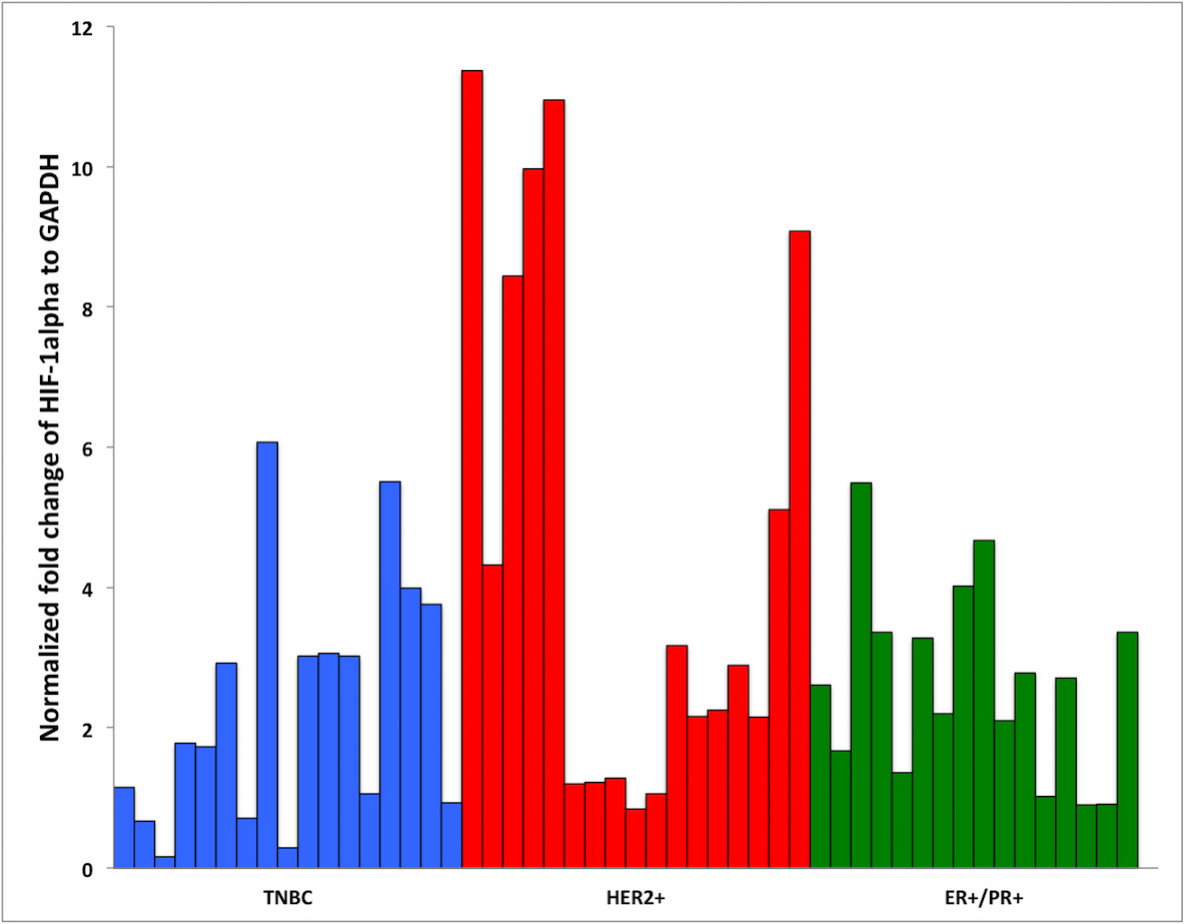
HIF-1α fold increase mRNA expression in TNBC, HER2+, ER+/PR+.

The quantity of extracted RNA ranged from 8 to 1053 ng/μl (mean = 264 ng/μl ± SD = 233 ng/μl, SEM = 32 ng/μl). Only 4 specimens yielded an RNA concentration < 65 ng/μl. These four cases were excluded. The variability in the quantity of extracted RNA may have resulted from the physical dimensions of the embedded tissue (biopsy versus resection) and nature of the specimen (fatty or fibrous tissues resulted in relatively lower RNA yields). All RNA samples gave a 260/280 nm ratio of ~2.0. qRT-PCR analysis results correlated with the immunohistochemical expression profiles of the studied biomarkers. The mean fold changes in the HIF-1α were 2.34,4.56, and 2.66for the TNBC, HER2^+^, and ER^+^/PR^+^respectively. This finding was significantly higher in HER2^+^(p = 0.043) (Fig 1). The mean fold changes in VEGF were 1.87, 1.37and 1.58 for the respective groups and revealed no significant variation (p = 0.173) (Table 2).

**Table 2:** Molecular features of TNBC as compared to the HER2^+^ and ER^+^/PR^+^ breast carcinoma.

Molecular Features	TNBC	HER2^+^	ER^+^/PR^+^	P-value
**HIF-1α**				
IHC	35.50%	45.30%	25.00%	0.055
qRT-PCR*	2.34^▯^	4.56^▯^	2.66	0.03^▯^
**VEGF**				
qRT-PCR	1.87	1.37	1.58	0.173
**VEGFR3** *				
IHC	54.8%^❖^	68.8%^❖^	39.1%^❖^	0.003^❖^

* Indicates a significant difference at the 5% level

▯Indicate significant differences between TNBC and HER2^+^ groups

^❖^Indicate significant differences between TNBC, HER2^+^ and ER^+^/PR^+^ groups

**Fig 2:**
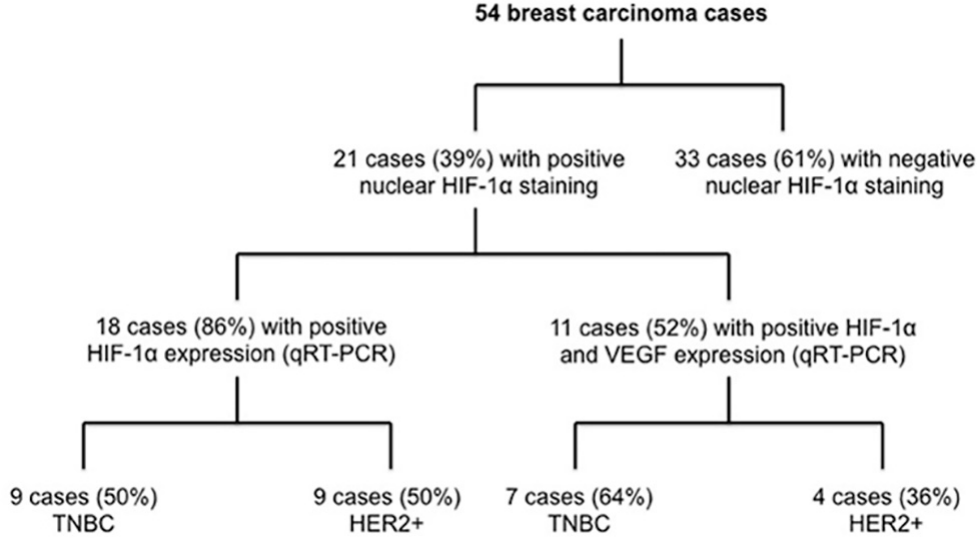
The percentage of TNBC and Her2 + cases with positivenuclearexpression of HIF-1α that expressed HIF-1α and VEGF transcripts fold change.

Transcriptional levels of HIF-1α and VEGF, and HIF-1α nuclear expression were correlated in the selected 50 cases. 19cases (38%) showed HIF-1α nuclear expression; 16/19 (84.2%) showed positive transcriptional levels of HIF-1α distributed equally between TNBC and HER2^+^. Moreover, 9/ 21 (40.9%) expressed both HIF-1α and VEGF transcripts. Of these 9 cases, 6 (66.7%) were TNBC cases and the remaining 3 (33.3%) were HER2^+^(Fig 2).

### Correlation of HIF-1β with HIF-1α expression in breast carcinoma cases

**Fig 3:**
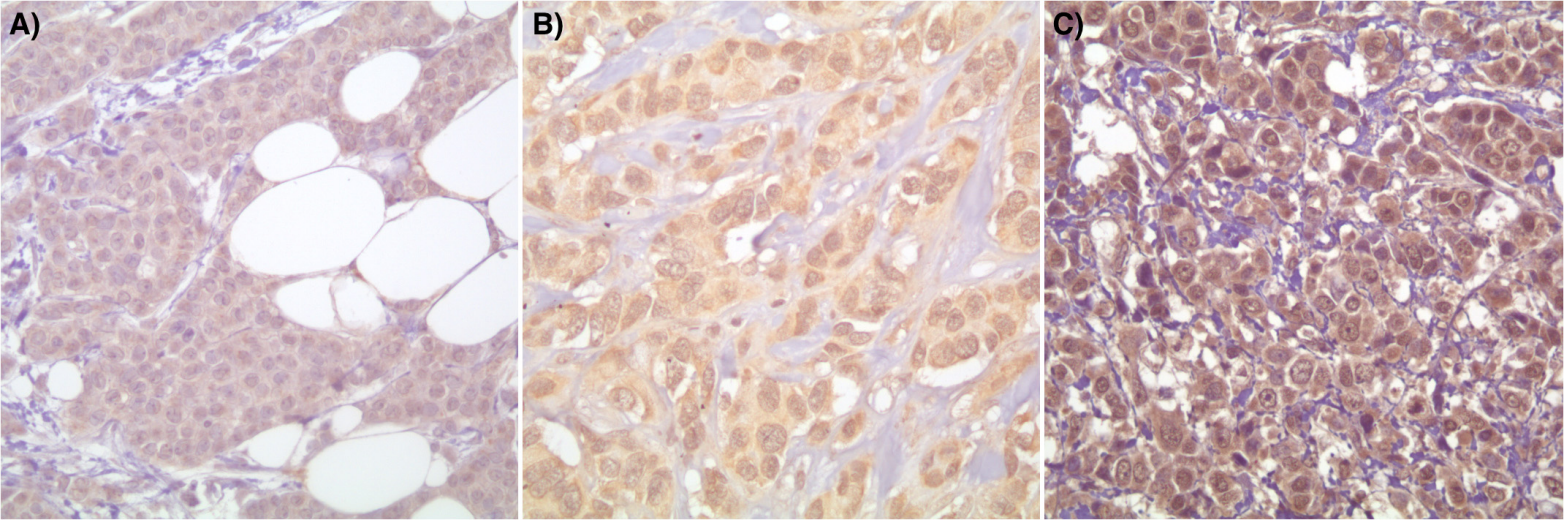
HIF-1αexpression in breast ductal carcinoma cells with negative (A) and weak (1+ and 2+) expression (B & C) (400x magnification).

HIF-1α immunoexpression was noted to be mostly weak staining (1+ and 2+) in 35/97 (36.1%) cases (Fig 3). This indicates that HIF-1α is not the sole contributor to the hypoxia driven angiogenesis. Therefore HIF-1β was assessed in order to determine whether the difference in HIF-1α positive cases is due to a difference in HIF-1β expression. Immunohistochemistry for HIF-1β protein was evaluated revealing that 31/97 (32.0%) cases were HIF-1β(+)/HIF-1α(+), 43/97 (44.3%) HIF-1β(-)/HIF-1α(+), 4/97 (4.1%) HIF-1β(+)/HIF-1α(-) and 19/97 (19.6%) HIF-1β(-)/HIF1α(-). When comparing HIF-1α expression with HIF-1β expression, the findings indicated a statistical difference between the status of HIF-1α expression and corresponding HIF-1β immunoexpression (p = 0.033; χ^2^ test).

**Fig 4:**
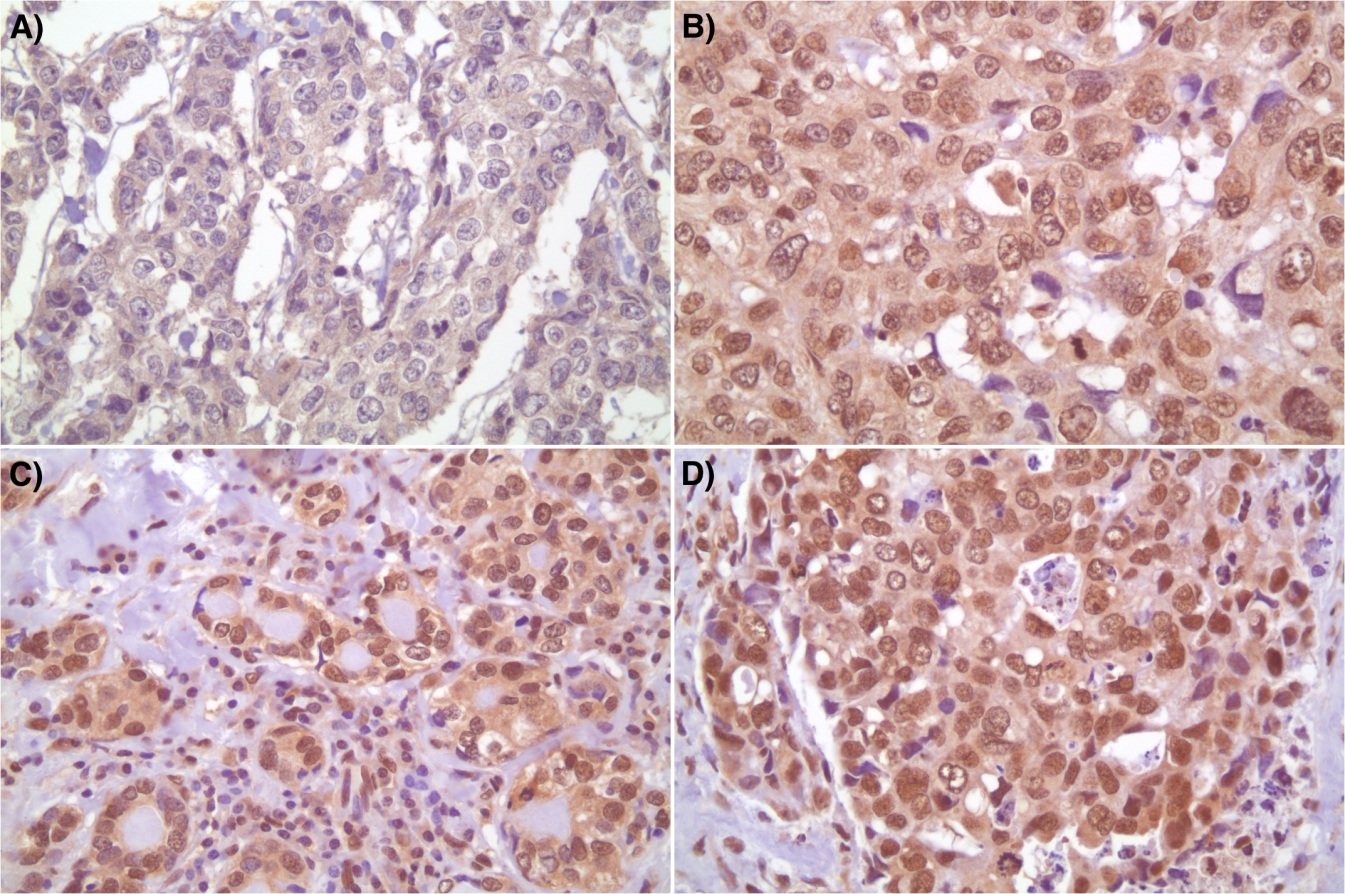
HIF-1β nuclear immunoexpression at various intensities showing negative staining (A), weak (1+) staining (B), moderate (2+) staining (C) and strong (3+) staining (D).

Furthermore, HIF-1α expression is known to stimulateVEGF expression[35]. The question is whether the difference in HIF-1α immunoexpression and VEGF mRNA expression correlates with HIF-1β immunoexpression (Fig 4, Table 3). In HIF-1β positive cases 13/42 (31.0%) VEGF tested cases were HIF-1α(+)/VEGF(+), 12/42 (28.6%) HIF-1α(-)/VEGF(+), 2/42 (4.8%) HIF-1α(+)/VEGF (-) and 4/42 (9.5%) HIF-1α (-)/VEGF (-). In HIF-1β negative cases, the status of HIF-1α and VEGF were as follows; 1/42 (2.4%) HIF-1α(+)/VEGF(+), 8/42 (19.1%) HIF-1α(-)/VEGF (+), 0/42 HIF-1α (+)/VEGF(-) and 6/42 (14.3%) HIF-1α(-)/VEGF(-) Table 4.Therefore, following correlation of HIF-1β status with HIF-1α in VEGF positive breast cancer revealed a statistically significant difference (p = 0.033; χ^2^ test). This implies that VEGF expression correlates with HIF-1α and HIF-1β expression. However, the question was whether a low VEGF expression correlates with a low HIF-1β and HIF-1α expression. The results were nonstatistically significant (p>0.05; χ^2^ test) implying that the findings are not reciprocal; note that the number of cases was low in VEGF negative cases (n = 8).

**Table 3:** Immunohistochemistry for HIF-1α and HIF-1β.

Immunoexpression	HIF-1α	HIF-1β
Negative	62/97 (63.9%)	23/97 (23.7%)
Weak	35/97 (36.1%)	50/97 (51.5%)
Strong	0/97	24/97 (24.7%)

HIF, Hypoxia inducible factor

**Table 4:** Correlation of HIF-1β with HIF-1α and VEGF.

HIF-1β	HIF-1α (+)/	HIF-1α (-) /	HIF-1α (+)/	HIF-1α (-)/
	VEGF (+) (%)	VEGF (+) (%)	VEGF (-) (%)	VEGF (-) (%)
Positive	13/42 (31.0)	12/42 (28.6)	2/42 (4.8)	4/42 (9.5)
Negative	1/42 (2.4)	8/42 (19.1)	0/42	6/42 (14.3)

HIF, Hypoxia Inducible Factor; VEGF, Vascular Endothelial Growth Factor

HIF-1α and HIF-1β were expressed in cases with central fibrosis and necrosis. Thehighest expression for HIF-1αwasin HER2^+^ followed by TNBC, while HIF-1β was expressed in 77% of TNBC; however no statistical significant correlation was observed and sample size was relatively small to draw a definite conclusion (Table 5).

**Table 5:** Correlation of Central Fibrosis with HIF-1α and HIF-1β expression.

Central Fibrosis	Overall (%)	HIF-1α	p-value	HIF-1β	p-value
			(HIF-1α)		(HIF-1β)
Present	29/190 (15.3)	16/29 (55.2)	p = 0.018*	7/9 (77.8)	p = 0.528
Absent	161/190 (84.7)	52/161 (32.3)		37/55 (67.3)	

HIF-1α, Hypoxia Inducible Factor-1 alpha; HIF-1β, Hypoxia Inducible Factor-1 beta

## Discussion

Hypoxia is a complex process associated with aggressive phenotype in many solid tumors including breast cancer[1,2]. A major regulator of hypoxia is the transcription factor HIF-1α. HIF-1α is a part of a heterodimeric protein that undergoes, under normoxicconditions, post-translational ubiquitination followed by proteosomal degradation. During hypoxia, this reaction is inhibited and HIF-1α is stabilized and dimerizes with its constitutive counterpart HIF-1β, forming a complex that translocates to the nucleus, and binds to the hypoxia response elements[36]. This complex activates the transcription of genes involved in cell growth, cell survival, and angiogenesis, consequently facilitating tumor progression and metastasis[4,37]. Identifying potential targets for anti-HIF-1α treatment among breast tumors is an appealing goal, especially for tumors such as TNBC which, as of yet, have no available targeted therapy. We therefore investigated the relative expression of HIF-1α and related angiogenic factors among the three main groups of breast cancer listed above. Our results revealed that TNBC, contrary to expectation, differed only slightly and with little to no statistical significance from the other subgroups, and that HER2 positive tumors showed the highest levels of expression for all studied parameters.

The initial expectation that HIF-1α should be increased in TNBC comes from its high documented levels in hereditary *BRCA1* mutated carcinomas (up to 90% of cases)[10]. Given that BRCA1-associated breast cancers often belong to the TNBC subtype, and both frequently show morphologic evidence of hypoxia (central fibrosis and necrosis)[9,27,28,38], an augmented expression of HIF-1α in tumors with a triple-negative phenotype was anticipated. In fact, this had been elegantly demonstrated through the preferential expression of HIF-1α in peri-necrotic/peri-fibrotic tumor cells in TNBC and *BRCA1* mutated breast cancers[10,39]. In contrast Tan etal. and Choi et al demonstrated an increase in TNBC of CAIX (carbonic anhydrase IX), a downstream product of the hypoxic pathway, rather than an increase in HIF-1α per se[40,41]. The authors did not dispute the likely contribution of hypoxia to the tumors’ aggressive phenotype, however. Our findings seem to be in line with the latter authors’ findings.

In the case of HER2 amplified tumors where hypoxia is not a prominent histologic feature, HIF-1α appears to act in concert with HER2, contributing to aggressive tumor biology. HER2 is a transmembrane tyrosine kinase receptor whose overexpression in breast carcinoma is a major contributor to tumor progression and metastasis[42,43]. HER2 appears to stabilize HIF-1α under normoxic conditions through tyrosine kinase receptor activation, consequently promoting VEGF secretion[44,45]. Recently, Whelan et al. showed that HIF-1α plays a role in HER2 over-expression and oncogenesis by regulating anoikis[46]. Thus the increase in HIF-1α expression and HER2 over-expression may be synergistic rather than necessarily an end product of hypoxic conditions. Nevertheless, the presence of HIF-1α seems likely to contribute to the aggressive tumor phenotype, regardless of the mechanism of its increased expression.

The lack of significant difference between TNBC and the ER^+^ group is also surprising. ER^+^/PR^+^/HER2^-^ breast carcinomas are less aggressive when compared to the TNBC and HER2 amplified groups[47]. ER expression, although known to contribute to breast cancer proliferation, is primarily a marker of better differentiation and renders the tumor responsive to Tamoxifen therapy. In this setting, HIF-1α tends to downregulate ERα[48–51]thus contributing to resistance to Tamoxifen treatment and worsening prognosis[52].

Our results did reveal that the protein as well as the mRNA expression for HIF-1α were the lowest in this subgroup even when corrected for tumor grade (data not shown), as reported in the literature, but the difference from TNBC failed to meet statistical significance[53,54].

Irrespective of breast cancer subgroup, we similarly did not establish any correlation between HIF-1α expression and age, grade, lymph node status, or MVD, which, when elevated in breast cancer, is thought to indicate an aggressive phenotype[55]. In the TNBC group, the detected mean MVD count was higher in cases that expressed HIF-1α, but not significantly, and showed no correlation with VEGF transcript fold increase. We could show a marginal correlation with VEGFR3 expression (p = 0.083), but with no special selectivity to TNBC.

However, HIF-1α expression appears to correlate with HIF-1β expression when positive. Similarly this is in conjunction with VEGF mRNA expression. Conversely, the absence of HIF-1α expression is inversely correlated with HIF-1β expression. With respect to VEGF expression, HIF1α positivity correlates with a HIF-1β expression. In addition, HIF-1β expression appears to correlate with VEGF status as expected[36]. Finally, HIF-1α and HIF-1β were mainly expressed in HER2 ^+^ and TNBC in cases with central fibrosis and necrosis without any statistical difference. Similar findings were demonstrated by Bos et al. that showed a higher expression of HIF1α, with the presence of necrosis in invasive breast cancer[48].

Based on these findings, the prospects of using anti-HIF-1α therapy is not likely to favor TNBC over other tumor groups, however, targeting HIF-1α may still prove beneficial given its definite expression in a significant portion of all studied breast cancer subtypes. This may be especially important in tumors that manifest aggressive clinical behavior. HIF-1α inhibitors are currently available but they do not exclusively target the HIF-1 pathway; and their efficacy in cancer therapy has not yet been established[56]. One clinical trial is currently recruiting patients with breast cancer to receive digoxin prior to surgery to block HIF-1α and potentially thwart cancer cell growth[57]. Large prospective trials with more specific agents will have to be undertaken to study the potential clinical use of this group of therapeutic agents in all breast cancer categories, not just TNBC.

This study is limited by the number of cases of each group, particularly in determining the mRNA expression of HIF-1α and VEGF (n = 18). Moreover, 85% of the TNBC while only 55% of ER+/PR+ of the cases o were grade 3, this factor may be have influence the results, however up to 95% of ER+/PR+ are less than grade 3 [58].

In summary, this study demonstrates that a proportion of TNBC is associated with hypoxia-related markers, that this association is not exclusive to TNBC but equally, if not more prominently, present in other breast cancer subtypes such as the HER2^+^ tumors, and finally that the presence of central fibrosis and necrosis correlate with higher HIF-1α expression levels in the studied cases. Although these findings do not identify a target that is specific for TNBC over other breast cancer subtypes, they do confirm the expression of high levels of HIF-1α at the transcriptional and protein levels in a variety of breast tumors, which may benefit from such targeted therapy, especially in the setting of clinically aggressive and drug resistant disease.
